# Cardiovascular Family History Increases the Risk of Disease Recurrence After a First Myocardial Infarction

**DOI:** 10.1161/JAHA.121.022264

**Published:** 2021-11-30

**Authors:** Agnes Wahrenberg, Ralf Kuja‐Halkola, Patrik K. E. Magnusson, Henrike Häbel, Anna Warnqvist, Kristina Hambraeus, Tomas Jernberg, Per Svensson

**Affiliations:** ^1^ Division of Cardiology Department of Clinical Science and Education Karolinska Institutet Södersjukhuset Stockholm Sweden; ^2^ Department of Medical Epidemiology and Biostatistics Karolinska Institutet Stockholm Sweden; ^3^ Karolinska Institutet Institute of Environmental Medicine Stockholm Sweden; ^4^ Department of Cardiology Falun Hospital Falun Sweden; ^5^ Department of Clinical Sciences Karolinska Institutet Danderyd University Hospital Stockholm Sweden

**Keywords:** ASCVD, family history, myocardial infarction, risk prediction, secondary prevention, SWEDEHEART, Cardiovascular Disease, Epidemiology, Risk Factors, Secondary Prevention

## Abstract

**Background:**

Family history of atherosclerotic cardiovascular disease (ASCVD) is easily accessible and captures genetic cardiovascular risk, but its prognostic value in secondary prevention is unknown.

**Methods and Results:**

We followed 25 615 patients registered in SWEDEHEART (Swedish Web‐System for Enhancement and Development of Evidence‐Based Care in Heart Disease Evaluated According to Recommended Therapies) from their 1‐year revisit after a first‐time myocardial infarction during 2005 to 2013, until December 31, 2018. Data on relatives, diagnoses and socioeconomics were extracted from national registers. The association between family history and recurrent ASCVD was studied with Cox proportional‐hazard regression, adjusting for risk factors and socioeconomics. A family history of ASCVD was defined as hospitalization due to myocardial infarction, angina with coronary revascularization, stroke, or cardiovascular death in ≥1 parent or full sibling, with early‐onset defined as disease‐onset before 55 years in men and 65 in women. The additional discriminatory value of family history to Thrombolysis in Myocardial Infarction Risk Score for Secondary Prevention was assessed with Harrell’s C‐index difference and reclassification was studied with continuous net reclassification improvement. Family history of early‐onset ASCVD in ≥1 first‐degree relative was present in 2.3% and was associated with recurrent ASCVD (hazard ratio [HR] 1.31; 95% CI, 1.17–1.47), fully adjusted for risk factors (HR, 1.22; 95% CI, 1.05–1.42). Early‐onset family history improved the discriminatory ability of the Thrombolysis in Myocardial Infarction Risk Score for Secondary Prevention, with Harrell’s C improving 0.003 points (95% CI, 0.001–0.005) from initial 0.587 (95% CI, 0.576–0.595) and improved reclassification (continuous net reclassification improvement 2.1%, *P*<0.001).

**Conclusions:**

Family history of early‐onset ASCVD is associated with recurrent ASCVD after myocardial infarction, independently of traditional risk factors and improves secondary risk prediction. This may identify patients to target for intensified secondary prevention.

**Nonstandard Abbreviations and Acronyms**
GRS: genetic risk score
rASCVD: recurrent atherosclerotic cardiovascular disease
SWEDEHEART: Swedish Web‐System for Enhancement and Development of Evidence‐Based Care in Heart Disease Evaluated According to Recommended Therapies

**Clinical Perspective**

**What Is New?**

- In this observational analysis from the nationwide myocardial infarction registry in Sweden, SWEDEHEART (Swedish Web‐System for Enhancement and Development of Evidence‐Based Care in Heart Disease Evaluated According to Recommended Therapies), we found that patients with a register‐verified family history of early‐onset atherosclerotic cardiovascular disease (ASCVD) in at least 1 first‐degree relative have a higher risk of recurrent ASCVD events after a first‐time myocardial infarction.
- Specifically, we found that patients with a family history of early‐onset ASCVD in at least 1 first‐degree relative had a 22% increased risk of recurrent ASCVD after a first myocardial infarction independently of other traditional risk factors, as compared with patients without such family history.
- The addition of family history of early‐onset ASCVD in at least 1 first‐degree relative improved the discriminatory ability of a validated risk prediction score for recurrent ASCVD, which may improve risk prediction in a secondary prevention setting, where intrinsic risk is already high.

**What Are the Clinical Implications?**

- The distinction of patients with a high‐risk profile in the stable setting after a myocardial infarction is important when identifying candidates for the most intensive secondary preventive treatment.
- Although genetic testing is not routinely feasible, patient‐derived family history is easily available in a clinical setting and captures both relevant genetic and environmental influences on disease risk.
- The findings of this study underline the importance of family history as a practical risk factor that improves risk stratification for future events also in patients with already prevalent ASCVD.

Coronary heart disease (CHD) is the leading cause of death worldwide. Although survival rates have improved alongside medical advances in treatment and secondary prevention measures, recurrent coronary events are common. In recent years, several genetic risk scores (GRS) incorporating single nucleotide polymorphisms associated with CHD in genome‐wide studies have been developed. It has been shown that a GRS based on the most common single nucleotide polymorphisms associated with CHD may identify patients with a higher risk of both incident and recurrent coronary events^1^, ^2^, ^3^ and that the genetic risk associated with CHD is independent of traditional risk factors.^4^ It has also been shown that the responsiveness to low‐density lipoprotein cholesterol (LDL‐C)‐lowering therapies with both statins and proprotein convertase subtilisin/kexin type‐9 inhibitors is greater in patients with high genetic risk,^1^, ^4^, ^5^ which may identify patients favored by intensified secondary preventive measures. Several attempts have been made to develop simple and ready‐at‐hand risk prediction models for recurrent cardiovascular events; however, the diagnostic precision has varied.^6^, ^7^, ^8^, ^9^ Family history of cardiovascular disease (CVD) may represent both genetic and environmental traits in families and it is often easily accessible information that may aid clinicians in adequate risk stratification of patients. A family history of myocardial infarction (MI) in a first‐degree relative is a marker for increased risk of a first‐time MI.^10^, ^11^ However, to what extent a family history of CVD carries prognostic information on recurrent cardiovascular events in a secondary prevention setting is unknown. Therefore, we sought to evaluate whether a family history of atherosclerotic CVD (ASCVD) is associated with recurrent cardiovascular events (rASCVD) in patients with a first‐time MI. Additionally, we aimed to study whether any such association was independent of traditional cardiovascular risk factors known to be genetically influenced, such as LDL‐C, as well as environmental factors including socioeconomic status (SES). Finally, we sought to study whether the addition of family history of ASCVD to a validated risk score would improve prediction of rASCVD events in survivors of MI.

## Methods

### Study Design and Data Sources

This was a nationwide, register‐based cohort study of patients with a first‐time MI, registered in the SWEDEHEART (Swedish Web‐system for Enhancement and Development of Evidence‐based care in Heart disease Evaluated According to Recommended Therapies) national quality registry. All hospitals in Sweden with acute cardiac care report to SWEDEHEART and the median coverage of reported MIs as compared with the Swedish National Patient Register was 96% in 2019.^12^ Until 2018, only patients aged ≤75 years at the time of the MI were enrolled in the longitudinal secondary prevention part of the registry. Clinical data at the time of the index MI and at a standardized 1‐year follow‐up in SWEDEHEART were cross‐referenced to Swedish national registers by means of personal identification numbers issued to all Swedish residents by the Swedish Tax Agency. Because of the sensitive nature of the data collected for this study as part of the SWEDEHEART registry, data cannot be made available to other researchers. Biological relatives of index patients were identified via the Swedish Multi‐Generation Register, providing data on relatives of most Swedish individuals born in 1932 and onwards, resident in Sweden.^13^ Data on diagnoses of hospital admissions and causes of death of index patients until December 31, 2018, were obtained from the National Patient Register and the Cause of Death Register. Data on hospital admissions and causes of death of relatives were retrieved until December 31, 2014. Socioeconomic data were obtained from Statistics Sweden. The linkage process has been described elsewhere.^14^ The study was approved by the Regional Ethical Review Board in Stockholm (registration number 2015/124‐31/4).

### Study Cohort

We included all patients aged 18–76 years with a first‐time MI, registered in SWEDEHEART during 2005 and 2013, attending the 1‐year revisit of a standardized follow‐up program 1 year after the MI. Patients with a history of MI, ischemic stroke, coronary artery bypass grafting, or any percutaneous coronary intervention procedure before the index MI were excluded. Index patients with any identifiable relative in the Swedish Multi‐Generation Register were included for further analyses. The exclusion process is visualized in Figure S1.

### Exposure

A family history of ASCVD was defined as the occurrence of a register‐verified hospitalization or death due to MI, any coronary revascularization procedure, or ischemic stroke in first‐degree relatives, as registered before the MI in the index patient. First‐degree relatives were defined as parents or full siblings. Parental family history was defined as disease in at least 1 parent, and sibling family history was defined as disease in at least 1 full sibling. The primary exposure, family history of early‐onset ASCVD, was defined as disease onset before 55 years in male and 65 years in female relatives, respectively. Family history was assessed with a composite ASCVD definition as well as by CHD and stroke, individually. In addition, the occurrence of venous thromboembolic disease (VTE), defined as pulmonary embolism or deep vein thrombosis, in relatives was assessed separately with an age limit for early onset disease of 65 years. The corresponding codes according to the *International Classification of Diseases, Tenth Revision* (*ICD‐10*) and historical ICD codes used are provided in Table S1.

### Clinical Data

Baseline data on index patients collected from SWEDEHEART quality registers were age, sex, year of revisit after MI, smoking status (never, former, current), body mass index (BMI), estimated glomerular filtration rate as estimated from serum creatinine using the Chronic Kidney Disease Epidemiology Collaboration equation, previously diagnosed congestive heart failure, hypertension, diabetes, and inpatient data on serum lipids at the time of the index MI. LDL‐C was calculated from total cholesterol, high‐density lipoprotein cholesterol, and triglycerides using the Friedewald formula when a direct measurement was not available. Records of peripheral artery disease at the time of the index MI were retrieved from the National Patient Register corresponding to *ICD‐10* I702, I702A, I702C, I702X, I739, and I739B. Data on SES included information on disposable income in the year preceding the MI, categorized into quintiles stratified by sex and inclusion year, educational level (<10, 10–12, and >12 years), and marital status at the time of index MI.

### Outcome Definitions

The primary outcome was a composite of rASCVD, defined as a recurrent fatal or nonfatal MI, unstable angina pectoris requiring urgent revascularization, fatal and nonfatal ischemic stroke, and cardiovascular death registered in the National Patient Register or the Cause of Death Register during the follow‐up period. The *ICD‐10* coding used to classify the outcome is provided in Table S2. Last date of follow‐up was December 31, 2018.

### Statistical Analysis

Baseline characteristics were reported according to types of family history and categorical variables as frequencies and percentages and as medians and interquartile ranges for continuous variables. The association between parental, sibling, and any first‐degree relative history and the composite primary outcome was estimated with Cox proportional‐hazard regression models, with time from the 1‐year revisit as the underlying time scale. Model I was adjusted for age by means of a restricted cubic spline with 4 knots, sex and year of the 1‐year revisit. The mean age of parents at the birth of the index patient was also included as a covariate to adjust for age‐related differences in exposure of ASCVD between families. Model II was additionally adjusted for hypertension, diabetes, and smoking status at the time of the index MI. For a subgroup of patients for whom data on LDL‐C and BMI were available, a model II was additionally adjusted for LDL‐C and BMI. This model was also run with multiple imputation of missing values of LDL‐C and BMI; however, because results did not differ significantly, only the unimputed estimates were ultimately used. Model III was additionally adjusted for SES measures. In order to describe the association between family history and the outcome in subgroups of postinfarction treatments, the fully adjusted model was run in subgroups according to whether or not patients were on acetylsalicylic acid and statin treatment, as reported by the caregiver in SWEDEHEART. There were 541 clusters with 2 related individuals, 11 clusters with 3 related individuals, and a total of 24 500 unrelated individuals included, respectively, in the final cohort. Hazard ratios (HR) are presented with a 95% CI based on cluster robust standard errors to account for nonindependent observations in family clusters. Kaplan‐Meier survival curves were constructed to illustrate the probability of rASCVD‐free survival during follow‐up. Additionally, a fourth model was adopted from the externally validated Thrombolysis in Myocardial Infarction Risk Score for Secondary Prevention model^6^, ^15^, ^16^ in which the risk of a recurrent cardiovascular event is estimated by the occurrence of 9 different clinical features of patients with MI. To evaluate the predictive effect of family history of ASCVD in the model, all definitions of family history were individually added to the clinical features applicable from the original model: chronic heart failure, hypertension, diabetes, estimated glomerular filtration rate below 60 mL/min per 1,73 m^2^, current smoking, peripheral artery disease, and age >75 years at the 1‐year revisit. The effect on discriminative ability of the model by adding family history was estimated by calculating the differences in Harrell’s C‐index between models. Harrell’s C‐index is based on the correlation between survival time and prognostic information and gives a probability of correctly predicting which of 2 randomly chosen individuals would live longer.^17^ Furthermore, the effect of family history on reclassification and risk difference prediction was estimated with continuous net reclassification improvement, which sums 4 probabilities for a new model.The first is the increase in risk when an event has occurred and the decrease in risk when an event has not occurred, which describes the overall improvement of the new model. From this is deducted the decrease in risk when an event has occurred and the increase in risk when an event did not occur, which in turn describes when the new model performed less well than the original model.^18^ The integrated discrimination improvement was also calculated. It is estimated based on 2 further differences of the integrals over all possible cutoff values between 0 and 1 of sensitivity and 1 – specificity, respectively.^19^ In other words, it gives the difference in average sensitivity minus the difference in average 1 – specificity. The significance level was set at an alpha of 0.05 and was not adjusted for multiple testing. Analyses were performed with STATA (Stata 15, StataCorp LLC, College Station, TX) and R (Version 4.0.3, R Foundation for Statistical Computing, Vienna, Austria).

## Results

### Patient Characteristics

In total, 25 615 patients were enrolled in the cohort, of whom 3971 (15.5%) experienced rASCVD during a mean follow‐up time of 7.2 years. A history of early‐onset and ever‐occurring ASCVD in any first‐degree relative occurred in 1668 (6.5%) and 10 113 (39.5%) respectively. Family history of early‐onset and ever‐occurring ASCVD in parents were present in 593 (2.3%) and 8667 (33.8%), respectively. A sibling history of early‐onset and ever‐occurring ASCVD was present in 1097 (4.3%) and 2246 (8.7%), respectively. Baseline characteristics of subjects according to family history status are presented in Table 1. Patients with a family history of early‐onset ASCVD in any first‐degree relative were significantly younger and were more likely to be current smokers at the time of the index MI. Cardiovascular comorbidity such as diabetes and peripheral artery disease were significantly more common in subjects with a family history of early‐onset ASCVD in a first‐degree relative as compared with patients without such history.

**Table 1:** Baseline Characteristics of Patients According to Early‐Onset or Any Family History of ASCVD in Any First‐Degree Relative

	Family history of early ASCVD in ≥1 first‐degree relative			Family history of any ASCVD in ≥1 first‐degree relative		
	No N=23 947	Yes N=1668	*P* value	No N=15 502	Yes N=10 113	*P* value
Age, y, median (IQR)	64.40 (58.10–69.60)	60.90 (53.50–67.00)	<0.001	64.80 (58.00–70.20)	63.40 (57.70–68.40)	<0.001
Male sex, n (%)	17 549 (73.3%)	1185 (71.0%)	0.046	11 274 (72.7%)	7460 (73.8%)	0.066
Hypertension, n (%)	9053 (37.8%)	650 (39.0%)	0.34	5838 (37.7%)	3865 (38.2%)	0.31
Missing	153 (0.6%)	11 (0.7%)		88 (0.6%)	76 (0.8%)	
Diabetes, n (%)	3018 (12.6%)	246 (14.7%)	0.011	1974 (12.7%)	1290 (12.8%)	0.96
Missing	4 (0.0%)	0 (0.0%)		3 (0.0%)	1 (0.0%)	
Smoking, n (%)			<0.001			<0.001
Never	8601 (35.9%)	522 (31.3%)		5638 (36.4%)	3485 (34.5%)	
Former	7861 (32.8%)	517 (31.0%)		5069 (32.7%)	3309 (32.7%)	
Current	7388 (30.9%)	626 (37.5%)		4726 (30.5%)	3288 (32.5%)	
Missing	97 (0.4%)	3 (0.2%)		69 (0.4%)	31 (0.3%)	
Body mass index, n (%)			<0.001			0.95
<18.5	135 (0.6%)	8 (0.5%)		89 (0.6%)	54 (0.5%)	
18.5–25	6083 (25.4%)	382 (22.9%)		3921 (25.3%)	2544 (25.2%)	
25–30	9793 (40.9%)	650 (39.0%)		6303 (40.7%)	4140 (40.9%)	
>30	4869 (20.3%)	424 (25.4%)		3196 (20.6%)	2097 (20.7%)	
Missing	3067 (12.8%)	204 (12.2%)		1993 (12.9%)	1278 (12.6%)	
Low‐density lipoprotein cholesterol, median (IQR)	3.30 (2.63–3.99)	3.32 (2.65–4.04)	0.22	3.30 (2.63–3.99)	3.31 (2.63–4.00)	0.52
Disposable income, n (%)			0.003			<0.001
Quintile 1	3956 (16.5%)	321 (19.2%)		2685 (17.3%)	1592 (15.7%)	
Quintile 2	4614 (19.3%)	331 (19.8%)		3114 (20.1%)	1831 (18.1%)	
Quintile 3	4971 (20.8%)	336 (20.1%)		3180 (20.5%)	2127 (21.0%)	
Quintile 4	5120 (21.4%)	369 (22.1%)		3251 (21.0%)	2238 (22.1%)	
Quintile 5	5278 (22.0%)	311 (18.6%)		3264 (21.1%)	2325 (23.0%)	
Missing	8 (0.0%)	0 (0.0%)		8 (0.1%)	0 (0.0%)	
Education, n (%)			<0.001			0.011
<10 y	7861 (32.8%)	572 (34.3%)		5194 (33.5%)	3239 (32.0%)	
10–12 y	10 953 (45.7%)	821 (49.2%)		7009 (45.2%)	4765 (47.1%)	
>12 y	4927 (20.6%)	259 (15.5%)		3154 (20.3%)	2032 (20.1%)	
Missing	206 (0.9%)	16 (1.0%)		145 (0.9%)	77 (0.8%)	
Family status, n (%)			0.007			0.017
Married	13 856 (57.9%)	908 (54.4%)		8840 (57.0%)	5924 (58.6%)	
Missing	114 (0.5%)	9 (0.5%)		80 (0.5%)	43 (0.4%)	
Peripheral artery disease, n (%)	182 (0.8%)	24 (1.4%)	0.003	126 (0.8%)	80 (0.8%)	0.85
Estimated glomerular filtration rate, n (%)			<0.001			0.001
>=90	9755 (40.7%)	788 (47.2%)		6260 (40.4%)	4283 (42.4%)	
60–90	11 349 (47.4%)	702 (42.1%)		7350 (47.4%)	4701 (46.5%)	
30–60	1899 (7.9%)	108 (6.5%)		1264 (8.2%)	743 (7.3%)	
15–30	122 (0.5%)	5 (0.3%)		90 (0.6%)	37 (0.4%)	
<15	54 (0.2%)	4 (0.2%)		33 (0.2%)	25 (0.2%)	
Missing	768 (3.2%)	61 (3.7%)		505 (3.3%)	324 (3.2%)	

ASCVD indicates atherosclerotic cardiovascular disease; and IQR, interquartile range.

### Primary Outcome Analysis

HRs for rASCVD according to the different definitions of family history of ASCVD are presented in Figure and Table 2. Kaplan‐Meier curves illustrating the probability of rASCVD‐free survival during follow‐up, by family history of ASCVD, are presented in Figure S2. HRs with regard to family history of stroke, CHD, and VTE are presented in Table S3. A family history of ASCVD in at least 1 first‐degree relative was significantly associated with rASCVD regardless of the age of onset in relatives, and the association remained significant when adjusted for cardiovascular risk factors including LDL‐C, BMI, and SES. The association was stronger with regard to early‐onset ASCVD in at least 1 first‐degree relative, with a fully adjusted HR of 1.22 (95% CI, 1.05–1.42). For parental and sibling history of ASCVD, the associations were largely similar, however with wider CIs indicating statistical nonsignificance when adjusted for risk factors. In contrast, early‐onset stroke in any first‐degree relative or in any full sibling was significantly associated with rASCVD across all adjustment models with a fully adjusted HR of 1.44 (95% CI, 1.10–1.90). Additionally, a family history of VTE in any first‐degree relative was significantly associated with rASCVD only when fully adjusted for multiple risk factors, including BMI, LDL‐C, and SES (HR, 1.24; 95% CI, 1.03–1.50), with the predominant effect presumably deriving from parental history. However, an early presentation of VTE in any first‐degree relative was not associated with rASCVD. Fully adjusted HRs for rASCVD in subgroups of acetylsalicylic acid and statin treatments are presented in Table S4. Although all point estimates were positive, CIs were wider, and all HRs were nonsignificant.

**Table 2:** Hazard Ratios for rASCVD With 95% CIs, by Analysis Model and Type of Family History

Model	History in any first‐degree relative		Parental history	Sibling history
Family history of early‐onset ASCVD				
I		1.31 (1.17–1.47)	1.47 (1.21–1.79)	1.23 (1.07–1.41)
II		1.22 (1.05–1.42)	1.27 (0.97–1.65)	1.19 (0.99–1.43)
III		1.22 (1.05–1.42)	1.28 (0.99–1.67)	1.18 (0.99–1.42)
Family history of ASCVD, any age of onset				
I		1.10 (1.03–1.17)	1.09 (1.02–1.17)	1.08 (0.97–1.20)
II		1.09 (1.00–1.18)	1.08 (0.99–1.17)	1.11 (0.97–1.26)
III		1.10 (1.01–1.19)	1.09 (1.00–1.19)	1.11 (0.97–1.27)

I—basic model adjusted for age, sex, year of last follow‐up. II—additionally adjusted for hypertension, diabetes, smoking status, body mass index, and low‐density lipoprotein cholesterol. III—additionally adjusted for socioeconomic status. ASCVD indicates atherosclerotic cardiovascular disease; and rASCVD, recurrent atherosclerotic cardiovascular disease.

### Predictive Value of Family History of ASCVD

Overall, the Thrombolysis in Myocardial Infarction Risk Score for Secondary Prevention risk prediction model for rASCVD, as performed with the applicable clinical features in this cohort, showed poor discriminatory ability, with a Harrell’s C‐index of 0.587 (95% CI, 0.576–0.595). The addition of family history of ever‐occurring and early‐onset ASCVD in any first‐degree relative improved discriminatory ability, with a C‐index difference of 0.003 and 0.002, respectively (95% CI of 0.001–0.005 and 0.001–0.004, correspondingly). A family history of ever‐occurring CHD in at least 1 first‐degree relative or in a parent also improved discrimination (Harrell’s C‐index difference 0.003 [95% CI, 0.001–0.006] and 0.003 [95% CI, 0.000–0.005] respectively). The addition of any other family history, however, did not improve discrimination. With regard to reclassification and risk difference prediction, the addition of a family history of early‐onset ASCVD in any first‐degree relative resulted in a continuous net reclassification improvement of 2.1% (*P*<0.001) and an integrated discrimination improvement of 0.00074 (*P*<0.00001). A family history of early‐onset CHD in any first‐degree relative or in parents, as well as early‐onset stroke in first‐degree relatives and siblings, rendered small but statistically significant improvements in measures of integrated discrimination improvement and continuous net reclassification improvement. Other definitions of family history did not improve the Thrombolysis in Myocardial Infarction Risk Score for Secondary Prevention model. Measures of discrimination and risk difference prediction are presented in Table 3 and Table S5.

**Table 3:** Harrell’s C‐index Difference, IDI, and cNRIs According to Type of Family History Added to TRS2⁰P Model

	History in any first‐degree relative	Parental history	Sibling history
Family history of early‐onset ASCVD			
Harrell’s C‐index difference (95% CI)	0.002 (0.001 to 0.004)	0.001 (−0.001 to 0.003)	0.001 (−0.000 to 0.003)
cNRI (*P* value)	0.02088 (*P*<0.0001)	0.02095 (*P*=0.01597)	0.01023 (*P*=0.02395)
IDI (*P* value)	0.00074 (*P*<0.00001)	0.00049 (*P*=0.01198)	0.00028 (*P*=0.9980)
Family history of ASCVD, any age of onset			
Harrell’s C‐index difference (95% CI)	0.003 (0.001 to 0.005)	0.002 (−0.000 to 0.004)	0.001 (−0.000 to 0.003)
cNRI (*P* value)	0.01448 (*P*=0.13174)	0.00919 (*P*=0.24351)	0.00725 (*P*=0.15170)
IDI (*P* value)	0.00008 (*P*=0.36727)	0.00007 (*P*=0.46307)	0.00003 (*P*=0.53493)

ASCVD indicates atherosclerotic cardiovascular disease; cNRI, continuous net reclassification improvement^18^; IDI, integrated discrimination improvement; and TRS2⁰P, Thrombolysis in Myocardial Infarction Risk Score for Secondary Prevention.^19^

## Discussion

By combining national registers of survivors of MI, we have shown that a family history of early‐onset ASCVD is associated with rASCVD after a first‐time MI, independently of several well‐established cardiovascular risk factors, including LDL‐C, consequently known to be associated with ASCVD.^20^, ^21^ Furthermore, the association was independent of several measures of SES that previously have been associated with incident and recurrent ASCVD.^22^, ^23^ Although family history of ASCVD is a widely known risk factor for incident CVD,^10^, ^11^ its relevance in the secondary preventive setting has been largely unknown. In this study, we show that family history remains an important clinical risk factor for rASCVD in a patient population with a high cardiovascular burden. The addition of family history of early‐onset ASCVD to a validated secondary risk prediction tool rendered statistically significant improvements in measures of discrimination, risk difference prediction, and risk category reclassification, highlighting that family history may be of particular importance in distinction of individuals at an already towering risk of rASCVD. However, as absolute improvements and the overall performance of the Thrombolysis in Myocardial Infarction Risk Score for Secondary Prevention model were poor, further evaluations in unrelated samples are needed to strengthen clinical significance. These novel findings may aid physicians in risk prediction and patient selection for intensified therapy in a secondary prevention setting.

Since the identification of the first risk alleles for CHD during the past decade, several risk scores based on genetic determinants of cardiovascular disease have been proposed. GRSs based on the most common risk alleles for CHD have repeatedly shown positive associations with incident ASCVD^1^, ^2^, ^4^; however, the value of GRSs in the prediction of recurrent disease has been questioned. For instance, the 9p21 locus strongly associated with incident CHD was recently presented to be statistically unrelated to subsequent CHD events in patients with known disease.^24^ Similarly, the association between a polygenic risk score and acute coronary syndrome was found to be weaker in individuals with known stable angina pectoris as compared with individuals without known angina pectoris.^25^ In contrast, several GRSs have shown to be positively associated with rASCVD.^4^, ^26^, ^27^, ^28^ Importantly, the family history studied in this paper may represent a composite of both genetic and environmental familial effects on cardiovascular risk. Possibly, some of these familial clusters of early and recurrent ASCVD events may represent families with known or unknown familial hypercholesterolemia and other less known heritable dyslipidemias; however, this is unlikely to account alone for the observed effect as a strong association remained after adjusting for LDL‐C. Environmental risk factors for ASCVD may cluster within families beyond the expression of traditional metabolic risk factors adjusted for in this study, such as physical inactivity and dietary habits. Still, there may be yet unknown genetic factors that may influence familial risk. Several studies of genetic risk have uncovered that a high GRS for ASCVD may be mitigated by a healthy lifestyle, highlighting the importance of environmental influences on genetic risk.^29^ Furthermore, patients with high genetic risk appear to respond better to both statin treatments^1^, ^5^ as well as proprotein convertase subtilisin/kexin type‐9 inhibitors^4^, ^27^ in both primary and secondary prevention settings. Additionally, although techniques for genetic analysis are becoming increasingly inexpensive, the feasibility of such analyses in clinical practice is still limited. A detailed family history represents ready‐at‐hand information of such genetic and environmental composites, which may alter the scope of clinical application of GRS studies. Because of the high cost of new lipid‐lowering treatments and the practical hindrances of genetic risk mapping in clinical practice, a detailed family history may well substitute as an easy measure to risk stratify patients for intensified lipid‐lowering treatment.

As shown in this study, a family history of early‐onset stroke in siblings appears to be of special importance in risk mediation, which suggests a clinically important pathway between the heritability of stroke on overall incident and recurrent ASCVD. These findings may also suggest that shared environmental exposure between siblings could influence rASCVD heritability to a greater extent than the parental exposure.^30^, ^31^, ^32^ HRs for a sibling history of stroke were not as heavily attenuated after adjustment of metabolic risk factors as with a family history of CHD, of which the association was largely attenuated after risk factor adjustment. The observed association between a family history of CHD and rASCVD could largely be attributed to parental family history, perhaps partly represented by residuals of age. Consequently, the observed associations of any family history of ASCVD, regardless of age of onset in relatives, and rASCVD were considerably weaker than for early‐onset family history, presumably representing the strong effect of age on the risk of both incident and recurrent events. The heritability of ASCVD in terms of future disease prediction is likely of less importance in an elderly patient group, as the risk imposed by age and the accumulated effect of traditional risk factors for ASCVD attributed to unique environmental factors during a lifetime may be of greater value in this setting. Additionally, the associations between a family history of VTE and rASCVD were positive only when adjusting for risk factors and without any restrictions in age of onset, suggesting a more complex interplay between VTE, ASCVD, and metabolic risk factors.

### Strengths and Limitations

One of the main strengths of this study is that this nationwide sample of unselected patients with a first‐time presentation of ASCVD may largely represent clinical reality and may render a high external validity of our findings. Swedish national registers provide uniquely detailed data on relatives, diagnoses, and SES, enabling a high internal validity of exposures, outcomes, and most covariates. An external validation of the Swedish National Patient Register reported positive predictive values for most diagnoses around 85% to 95%, and above 90% for MI.^33^ However, this study also has several limitations. First, as only patients attending the 1‐year revisit after the index MI were included, patients with fatal disease recurrences within 1 year of the primary MI have been dropped off, which may potentially underestimate of the observed effect of family history on overall short‐ and long‐term prognosis. However, patients with chronic coronary syndromes represent a newly defined patient group^34^ susceptible to sudden destabilization and new coronary events. As our intention was to identify secondary risk predictors in this specific patient group, only stable postinfarction patients were included. Furthermore, a subset of patients had missing register data on risk factors, including BMI but most important LDL‐C, which were missing in 5811 patients. As statistical multiple imputation of values did not alter the analytical results to any major extent, only the original data were analyzed in these cases, comprising beyond 15 000 individuals. Third, several patients were excluded from analysis owing to the absence of registered relatives in the Multi‐Generation Register. In a previous investigation on family history of coronary artery disease in patients seeking the emergency room with chest pain,^14^ missing parental data in the Swedish Multi‐Generation Register largely was because of index patients having immigrated to Sweden, with an inherently increased prevalence of metabolic risk factors. This may possibly also lead to underestimates of the effect of family history on recurrence rates. Moreover, absolute values of improved diagnostics with the addition of family history as measured with Harrell’s C‐index were small. In this population with established coronary disease, a substantial share (15.5%) of patients experienced rASCVD during follow‐up. Presumably, as all included patients in this study had a very high intrinsic risk of subsequent ASCVD events, the distribution of rASCVD risks in this population might have been narrow enough to render only small incremental effects on C‐index difference, regardless of the positive isolated HRs of risk factors.^35^ Similarly to the interpretations of the leveling effect of stable angina pectoris on associations between a polygenic risk score and acute coronary syndrome as studied by Lee et al,^25^ we cannot exclude that the association between a family history of ASCVD and rASCVD observed in this paper could be mitigated owing to index event bias, that is, that the selection of individuals based on a prevalent disease may influence associations between previously established risk factors and disease recurrence.

### Conclusions

In conclusion, we have shown that a family history of early‐onset ASCVD in first‐degree relatives is an independent risk factor for rASCVD in survivors of MI and that the addition of family history information improved the performance of a validated risk prediction model for rASCVD. Our findings highlight the significance of family history as a traditional risk factor also in the secondary preventive setting. Although genetic testing is still not routinely available, family history is easily accessible in clinical practice and conveys both genetic and environmental influences on risk. Using family history in the risk assessment of these patients may help to identify those suitable for more intense secondary prevention measures, and in which new therapies can be efficient and cost effective.

## Sources of Funding

This work was funded by grants from The Swedish Heart and Lung Association.

## Disclosures

None.

**Figure 1:**
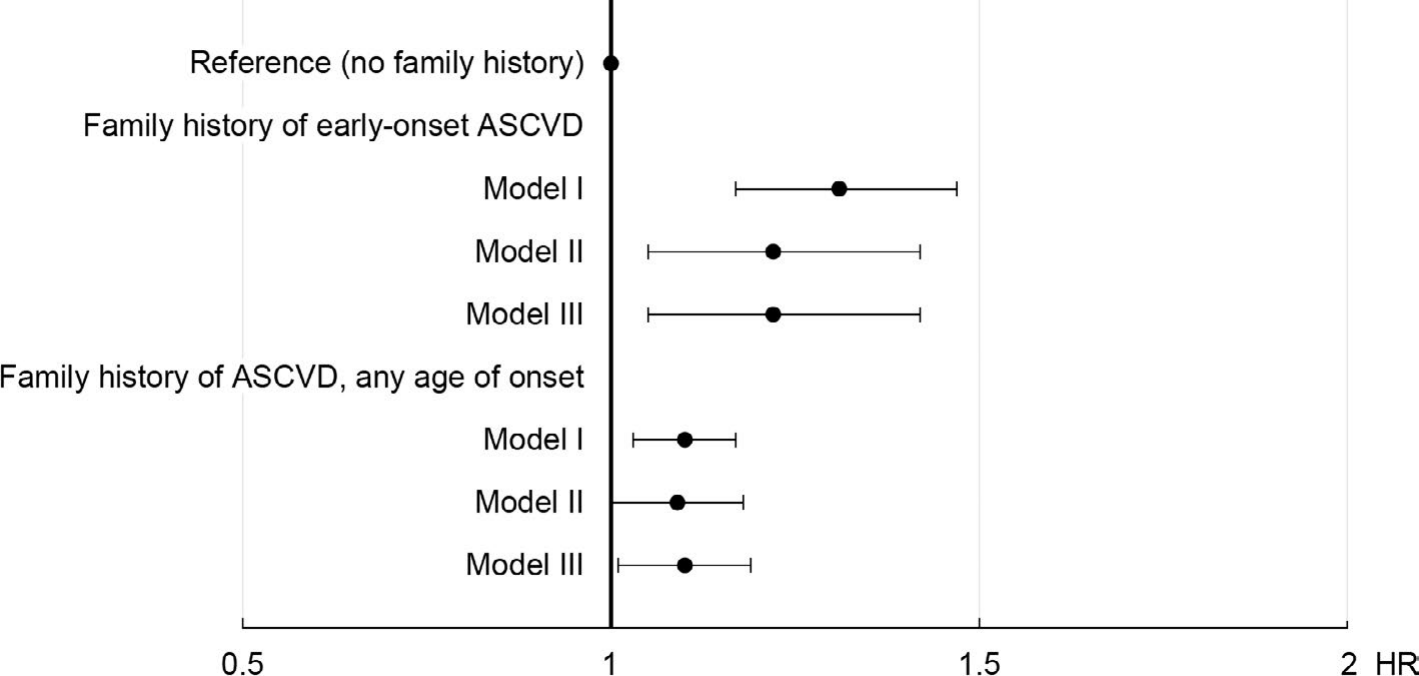
Hazard ratios for rASCVD according to family history of ASCVD in any first‐degree relative. Model I: basic model adjusted for age, sex, year of last follow‐up. Model II: additionally adjusted for hypertension, diabetes, smoking status, body mass index, and low‐density lipoprotein cholesterol. Model III: additionally adjusted for socioeconomic status. ASCVD indicates atherosclerotic cardiovascular disease; and rASCVD, recurrent atherosclerotic cardiovascular disease.

## Supporting information

Tables S1–S5
Figures S1–S2
