# Supplementary material for: Cardiovascular Family History Increases the Risk of Disease Recurrence After a First Myocardial Infarction
Source: J Am Heart Assoc. 2021 Nov 30;10(23):e022264. doi: 10.1161/JAHA.121.022264 (PMC9075368; doi:10.1161/JAHA.121.022264)
Supplement: Supplementary file 1 — Tables S1–S5 Figures S1–S2 [file JAH3-10-e022264-s001.pdf]

SUPPLEMENTAL MATERIAL

Table S1. Exposure definitions according to different ICD editions and Swedish procedure coding systems.

Familial disease of interest	*ICD-10*	*ICD-9*	*ICD-8*	*ICD-7*	*ICD-6*	*Swedish surgical procedure codes (KVÅ)*	*Historical surgical procedure codes (before 1997)*	*Register*
**Fatal or nonfatal myocardial infarction**	I21 – I22	410	410.00 – 410.99	4201	4201			Inpatient, Cause of death
**Coronary revascularization**						FNA, FNB, FNC, FND, FNE, FNF, FNG, FNW	3105, 3127, 3158, 3066, 3067, 3068, 3080, 3091	Inpatient
**Sudden cardiac death, cardiac arrest**	I461, I469	798B, 427F	795.99	795.2	795.2			Cause of death
**Fatal or nonfatal ischaemic stroke**	I630 – I635, I638 – I639	433 – 434, 436	432 – 434, 436	332	332			Inpatient, Cause of death
**Pulmonary embolism**	I260 – I269	415B	450.00 – 450.91, 450.03, 450.09	465	465			Inpatient, Cause of death
**Deep vein thrombosis**	I801 – 1803, I808 – I809	451B – 451C, 451W, 451X	451.98 – 451.99	463 – 464, 466	463 – 464, 466			Inpatient, Cause of death

ICD – International Classification of Diseases

Table S2. Outcome definitions according to ICD-10.

Outcome		ICD-10 code	Register used
**Nonfatal myocardial infarction**		I210 – I214, I219, I220 – I221, I228 – I229	Inpatient
**Unstable angina with coronary revascularization**		I20.0 with a simultaneous Swedish procedure code (KVÅ) of FNA, FNB, FNC, FND, FNE, FNF, FNG or FNW	Inpatient
**CHD death**	Fatal myocardial infarction	I210 – I214 & I219, I220 – 221, I228 – I229	Cause of death
	Sudden cardiac death	I469	Cause of death
**Nonfatal ischaemic stroke**		I630 – I635, I638 – I639	Inpatient
**Fatal ischaemic stroke**		I630 – I635, I638 – I639	Cause of death

CHD – coronary heart disease, ICD – International Classification of Diseases

Table S3. Hazard ratios (95% confidence intervals) for stroke, CHD and VTE, by analysis model and type of family history.

Family history of early-onset stroke			
**Model**	History in any first-degree relative	Parental history	Sibling history
**I**	1.54 (1.24 – 1.91)	1.48 (0.76 – 2.90)	1.56 (1.25 – 1.95)
**II**	1.46 (1.11 – 1.92)	1.31 (0.48 – 3.54)	1.47 (1.11 – 1.96)
**III**	1.44 (1.10 – 1.90)	1.30 (0.48 – 3.55)	1.46 (1.09 – 1.94)
Family history of stroke, any age of onset			
**Model**	History in any first-degree relative	Parental history	Sibling history
**I**	1.01 (0.93 – 1.10)	0.96 (0.87 – 1.06)	1.13 (0.99 – 1.29)
**II**	1.03 (0.93 – 1.14)	1.00 (0.88 – 1.13)	1.10 (0.93 – 1.30)
**III**	1.04 (0.93 – 1.15)	1.00 (0.88 – 1.13)	1.12 (0.95 – 1.32)
Family history of early-onset CHD			
**Model**	History in any first-degree relative	Parental history	Sibling history
**I**	1.23 (1.08 – 1.40)	1.45 (1.19 – 1.78)	1.10 (0.92 – 1.20)
**II**	1.15 (0.96 – 1.36)	1.26 (0.96 – 1.65)	1.08 (0.86 – 1.32)
**III**	1.15 (0.96 – 1.36)	1.28 (0.97 – 1.67)	1.07 (0.86 – 1.33)
Family history of CHD, any age of onset			
**Model**	History in any first-degree relative	Parental history	Sibling history
**I**	1.12 (1.05 – 1.20)	1.12 (1.04 – 1.20)	1.04 (0.90 – 1.22)
**II**	1.09 (1.00 – 1.19)	1.08 (0.98 – 1.18)	1.09 (0.90 – 1.32)
**III**	1.10 (1.01 – 1.21)	1.09 (1.00 – 1.20)	1.09 (0.90 – 1.32)
Family history of early-onset VTE			
**Model**	History in any first-degree relative	Parental history	Sibling history
**I**	0.94 (0.69 – 1.29)	1.19 (0.53 – 2.67)	0.90 (0.64 – 1.28)
**II**	1.00 (0.68 – 1.48)	0.98 (0.32 – 3.00)	1.00 (0.66 – 1.51)
**III**	0.99 (0.68 – 1.46)	0.92 (0.30 – 2.82)	1.00 (0.66 – 1.50)

Family history of VTE, any age of onset			
**Model**	History in any first-degree relative	Parental history	Sibling history
**I**	1.10 (0.94 – 1.28)	1.15 (0.95 – 1.39)	0.97 (0.73 – 1.28)
**II**	1.24 (1.03 – 1.50)	1.33 (1.07 – 1.66)	1.02 (0.72 – 1.44)
**III**	1.24 (1.03 – 1.50)	1.33 (1.07 – 1.66)	1.03 (0.73 – 1.45)

*I - basic model adjusted for age, gender, year of last follow-up. II – additionally adjusted for hypertension, diabetes mellitus, smoking status, BMI and LDL-c. III – additionally adjusted for SES.

† CHD – coronary heart disease, LDL-c – low-density lipoprotein cholesterol, SES – socioeconomic status, VTE – venous thromboembolism

Table S4. Fully adjusted* hazard ratios for rASCVD with 95% confidence interval, according to pharmacological post-infarction treatment and type of family history.

Family history of early-onset ASCVD				
	N	***History in any first-degree relative***	***Parental history***	***Sibling history***
On ASA	23,141	1.16 (0.98 – 1.37)	1.20 (0.90 – 1.62)	1.14 (0.94 – 1.39)
Not on ASA	1,663	1.41 (0.69 – 2.89)	1.71 (0.53 – 5.53)	1.06 (0.42 – 2.67)
On statin	22,783	1.14 (0.97 – 1.36)	1.18 (0.88 – 1.59)	1.12 (0.91 – 1.37)
Not on statin	1,994	1.35 (0.80 – 2.27)	2.00 (0.72 – 5.57)	1.19 (0.65 – 2.16)
Family history of ASCVD, any age of onset				
	N	***History in any first-degree relative***	***Parental history***	***Sibling history***
On ASA	23,141	1.08 (0.99 – 1.18)	1.08 (0.98 – 1.18)	1.05 (0.91 – 1.22)
Not on ASA	1,663	1.17 (0.84 – 1.64)	1.17 (0.82 – 1.66)	1.26 (0.70 – 2.28)
On statin	22,783	1.08 (0.98 – 1.18)	1.07 (0.98 – 1.18)	1.07 (0.92 – 1.24)
Not on statin	1,994	1.09 (0.84 – 1.42)	1.11 (0.84 – 1.47)	1.01 (0.64 – 1.58)

* Model adjusted for age, sex, year of last follow-up, hypertension, diabetes mellitus, smoking status, BMI, LDL-c and SES.

† ASA – acetylsalicylic acid, ASCVD – atherosclerotic cardiovascular disease, BMI – body mass index, LDL-c – low-density lipoprotein cholesterol, rASCVD – recurrent atherosclerotic cardiovascular disease, SES – socioeconomic status.

Table S5. Harrell's C-index difference, IDI and cNRIs according to type of family history added to the TRS2⁰P model.

Family history of early-onset stroke	*History in any first-degree relative*	*Parental history*	*Sibling history*
Harrell's C index difference (95% CI)	0.001 (-0.000 – 0.003)	0.000 (-0.000 – 0.000)	0.001 (-0.000 – 0.003)
cNRI (p-value)	0.00734 (p<0.00001)	0.03161 (p=0.63473)	0.00676 (p<0.00001)
IDI (p-value)	0.00054 (p=0.02794)	0.00006 (p=0.57086)	0.00051 (p=0.01996)
Family history of stroke, any age of onset	***History in any first-degree relative***	***Parental history***	***Sibling history***
Harrell's C index difference (95% CI)	-0.001 (-0.003 – 0.001)	0.000 (-0.001 – 0.002)	0.001 (-0.000 – 0.002)
cNRI (p-value)	0.00608 (p=0.29541)	0.01246 (p=0.14371)	0.00657 (p=0.11577)
IDI (p-value)	0.00005 (p=0.51098)	0.00016 (p=0.24750)	0.00012 (p=0.39521)
Family history of early-onset CHD	***History in any first-degree relative***	***Parental history***	***Sibling history***
Harrell's C index difference (95% CI)	0.001 (-0.000 – 0.003)	0.001 (-0.001 – 0.003)	0.001 (-0.000 – 0.001)
cNRI (p-value)	0.01409 (p=0.00399)	0.02010 (p=0.01198)	0.00424 (p=0.16367)
IDI (p-value)	0.00035 (p=0.03194)	0.00046 (p=0.01198)	0.00000 (p=0.51098)
Family history of CHD, any age of onset	***History in any first-degree relative***	***Parental history***	***Sibling history***
Harrell's C index difference (95% CI)	0.003 (0.001 – 0.006)	0.003 (0.000 – 0.005)	0.00 0 (-0.001 – 0.001)
cNRI (p-value)	0.02018 (p=0.01996)	0.01730 (p=0.05988)	0.00377 (p=0.21557)
IDI (p-value)	0.00020 (p=0.21557)	0.00012 (p=0.21956)	-0.00001 (p=1.00000)

* ASCVD – atherosclerotic cardiovascular disease, CI – confidence interval, cNRI – continuous net reclassification improvement[18], IDI – integrated discrimination improvement[19], TRS2⁰P - TIMI Risk Score for Secondary Prevention[15].

Figure S1. Flow chart of exclusion process.

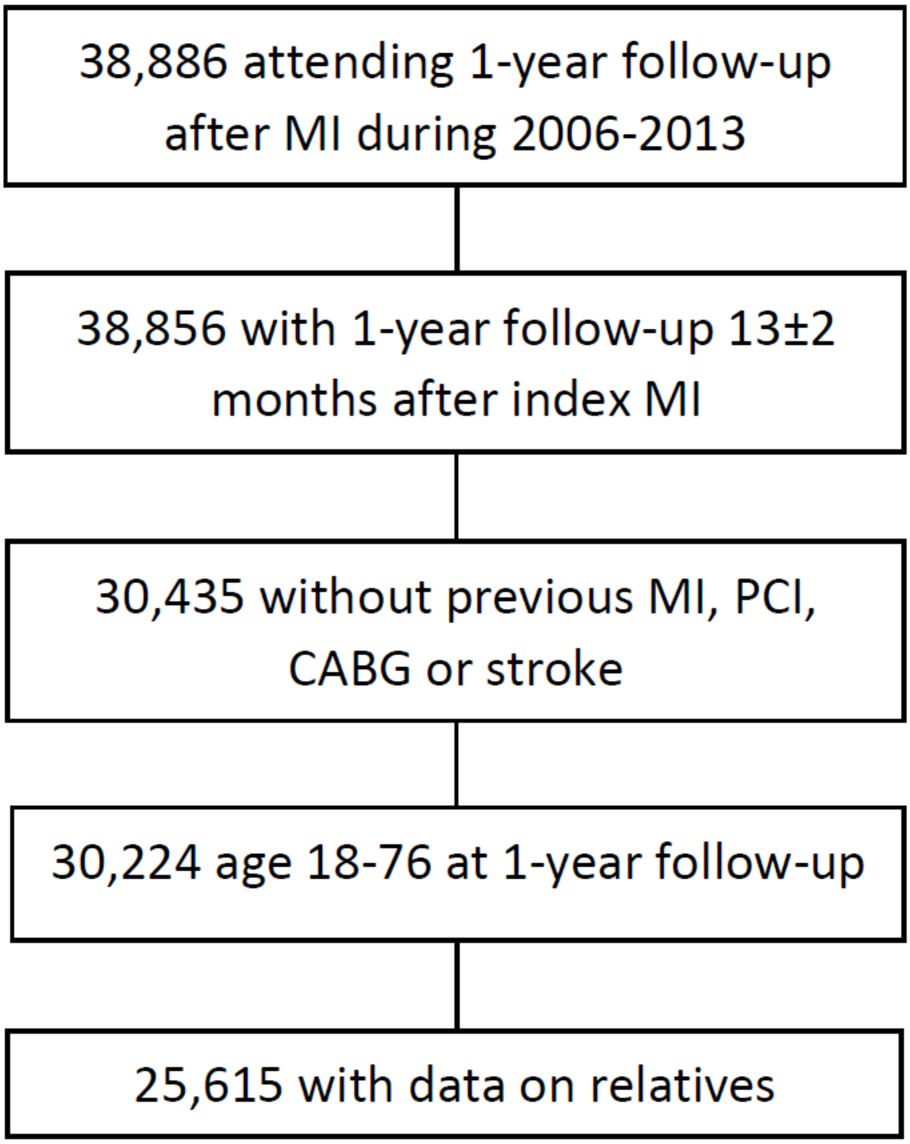

CABG – coronary artery bypass grafting, MI – myocardial infarction, PCI – percutaneous coronary intervention

Figure S2. Kaplan-Meier plots of outcomes over time in relation to family history status.

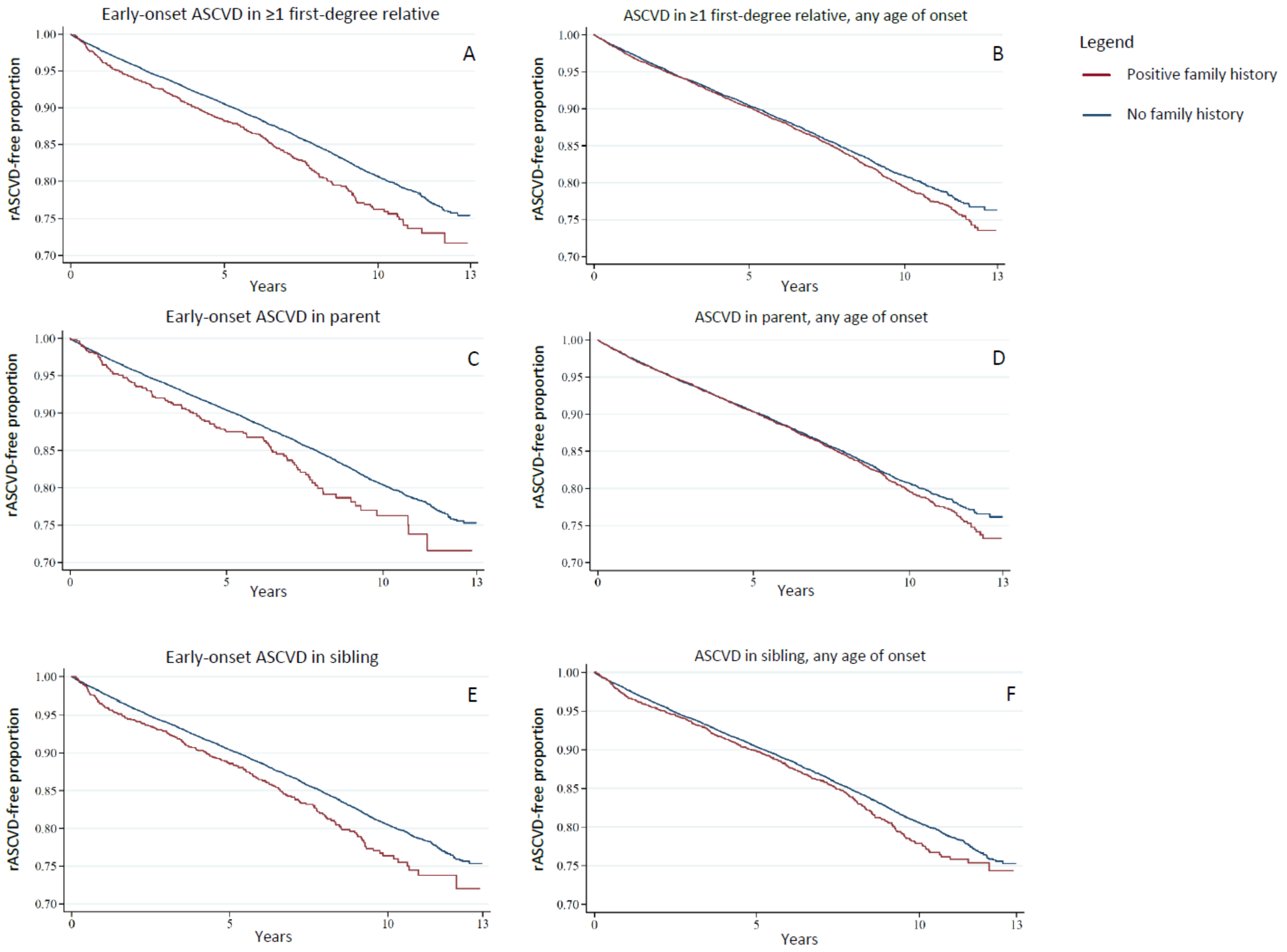

ASCVD – atherosclerotic cardiovascular disease, rASCVD – recurrent atherosclerotic cardiovascular disease
